# Alginate-amphotericin B nanocomplexes covered by nanocrystals from bacterial cellulose: physico-chemical characterization and in vitro toxicity

**DOI:** 10.1038/s41598-021-03264-1

**Published:** 2021-12-14

**Authors:** Victória Soares Soeiro, Ricardo Silva-Carvalho, Daniela Martins, Pier Parpot, Denise Grotto, Marco Vinicius Chaud, Francisco Miguel Portela da Gama, Angela Faustino Jozala

**Affiliations:** 1 LAMINFE – Laboratory of Industrial Microbiology and Fermentation Process, University of Sorocaba, Sorocaba, Brazil; 2 CEB - Centre of Biological Engineering, University of Minho, Campus de Gualtar, 4710-057 Braga, Portugal; 3 Centre of Chemistry, University of Minho, Campus de Gualtar, 4710-057 Braga, Portugal; 4 Lapetox – Laboratory of Toxicology Research, University of Sorocaba, Sorocaba, Brazil; 5 LABNUS – Biomaterials and Nanotechnology Laboratory, University of Sorocaba, Sorocaba, Brazil

**Keywords:** Biotechnology, Biomaterials, Nanobiotechnology

## Abstract

Nanocomplexes systems made up natural poylymers have pharmacotechnical advantages such as increase of water solubility and a decrease of drugs toxicity. Amphotericin B (AmB) is a drug apply as anti-leishmanial and anti-fungal, however it has low water solubility and high toxicity, limiting its therapeutic application. With this in mind, the present study aimed to produce nanocomplexes composed by alginate (Alg), a natural polymer, with AmB covered by nanocrystals from bacterial cellulose (CNC). For this reason, the nanocomplexes were produced utilizing sodium alginate, amphotericin B in a borate buffer (pH 11.0). The CNC was obtained by enzymatic hydrolysis of the bacterial cellulose. To CNC cover the nanocomplexes 1 ml of the nanocomplexes was added into 1 ml of 0.01% CNC suspension. The results showed an ionic adsorption of the CNC into the Alg-AmB nanocomplexes surface. This phenomena was confirmed by an increase in the particle size and PDI decrease. Besides, nanocomplexes samples covered by CNC showed uniformity. The amorphous inclusion of AmB complex into the polysaccharide chain network in both formulations. AmB in the nanocomplexes was in supper-aggregated form and showed good biocompatibility, being significantly less cytotoxic in vitro against kidney cells and significantly less hemolytic compared to the free-drug. The in vitro toxicity results indicated the Alg-AmB nanocomplexes can be considered a non-toxic alternative to improve the AmB therapeutic effect. All process to obtain nanocomplexes and it coat was conduce without organic solvents, can be considered a green process, and allowed to obtain water soluble particles. Furthermore, CNC covering the nanocomplexes brought additional protection to the system can contribut advancement in the pharmaceutical.

## Introduction

Amphotericin B (AmB) is a polyene antibiotic appliedas a gold standard therapy in fungal infections, because it does not cause microbial resistance^1^. The AmB is also applied as a second-line treatment for visceral leishmaniasis^2^. Despite being a drug widely used for more than half a century, AmB presents several limitations such as low water solubility and low permeability, in addition to high toxicity, especially nephrotoxicity^3^.

Nowadays, drug delivery systems have been used in order to overcome AmB limitations and improve its therapeutic efficacy. Thus, tifferent formulations containing AmB are commercialized such as AMBISOME and Abelcet®. These products are based on lipids, making an expensive formulations. Therefore, encapsulating AmB in a nanocomplex system composed by a natural polymer is an alternative found to solve the product problems^4^.

Nanocomplexes are self-organized structures composed by polymer (in many cases natural polysaccharides) and the drug. This system has advantages such as nano scale, high stability, good dispersion in water, low toxicity, with excellent cost–benefit and does not use solvents. In the literature, there are several works using different types of polymers, such as dextrin^5^, gum arabic^6^, albumin^7^ and alginate^8^. Alginate (Alg) is a polymer extracted from natural sources, widely used in the pharmaceutical area and in tissue engineering. This material has several advantages, such as immune system activation, nontoxicity, biocompatibility and biodegradability^9–11^. In our research, in order to reinforce and cover the system Alg-AmB, bacterial cellulose nanocrystals (CNC) were employed.

CNC can be used as a reinforcement material to drug delivery systems, protecting them and enabling the administration of the drug by different routes^12,13^. CNC are produced by the action of cellulase in contact with bacterial cellulose. This material has a renewable and sustainable origin, with good cost–benefit, biocompatibility and biodegradability, besides presenting a good mechanical resistance, large surface area, low toxicity^10,14,15^.

In this work, the novelties were produced the AmB nanocomplexes utilizing Alg and apply CNC to coat them. These CNC were produced by bacterial cellulose enzymatic hydrolyzed. For this reason, we have studied the influence of alginate in nanocomplex production and the CNC coat. Since, there is no delivery systems with Alg-AmB and CNC, it was evaluated the physical–chemical characterization and the in vitro toxicity.

## Results

### Physico-chemical characterization of nanocomplexes

Size, index of polydispersity (PDI) and zeta potential of the CNC, Alg-AmB nanocomplexes and the Alg-AmB nanocomplexes covered by CNC (Alg-AmB + CNC) are shown in Table 1.

**Table 1:** Formulation of alginate (Alg) and alginate-amphotericin B (Alg-AmB) to prepare the nanocomplexes.

Formulations	Borate buffer pH 11 (mL)	Sodium alginate (mg)	Amphotericin B (mg)
Alg	9.6	120	–
Alg-AmB	9.6	96	24

Observe the results in the Table 2, it is possible observe a significant increase of the Alg-AmB + CNC nanocomplex size, possibly due to coating by the CNC. On the other hand, the nanocomplexes with CNC the degree of uniformity (PDI) of the samples decreased. It means the Alg-AmB + CNC nanocomplexes showed a higher uniformity compared with sample without CNC.

**Table 2:** Analysis of the size, PDI and zeta potential of Alg-AmB Alg-AmB + CNC).

	CNC	Alg-AmB	Alg-AmB + CNC
Size (nm)	41.67 ± 5.55	258.87 ± 10.41	466.3 ± 17.57
Index of polydispersity	0.127 ± 0.005	0.523 ± 0.073	0.420 ± 0.05
Zeta potential (mV)	7.57 ± 2.26 mV	−62.93 ± 2.02	−55.75 ± 1.23

The CNC zeta potential value is positive due to enzymatic production and the Alg nanocomplexes zeta potential is negative, being as the best condition to cover with the CNC. In this way, observe the results in Table 2, the Alg-AmB + CNC showed a zeta potential higher than Alg-Amb. This increase was indicated the nanocomplexes coating.

**Figure 1:**
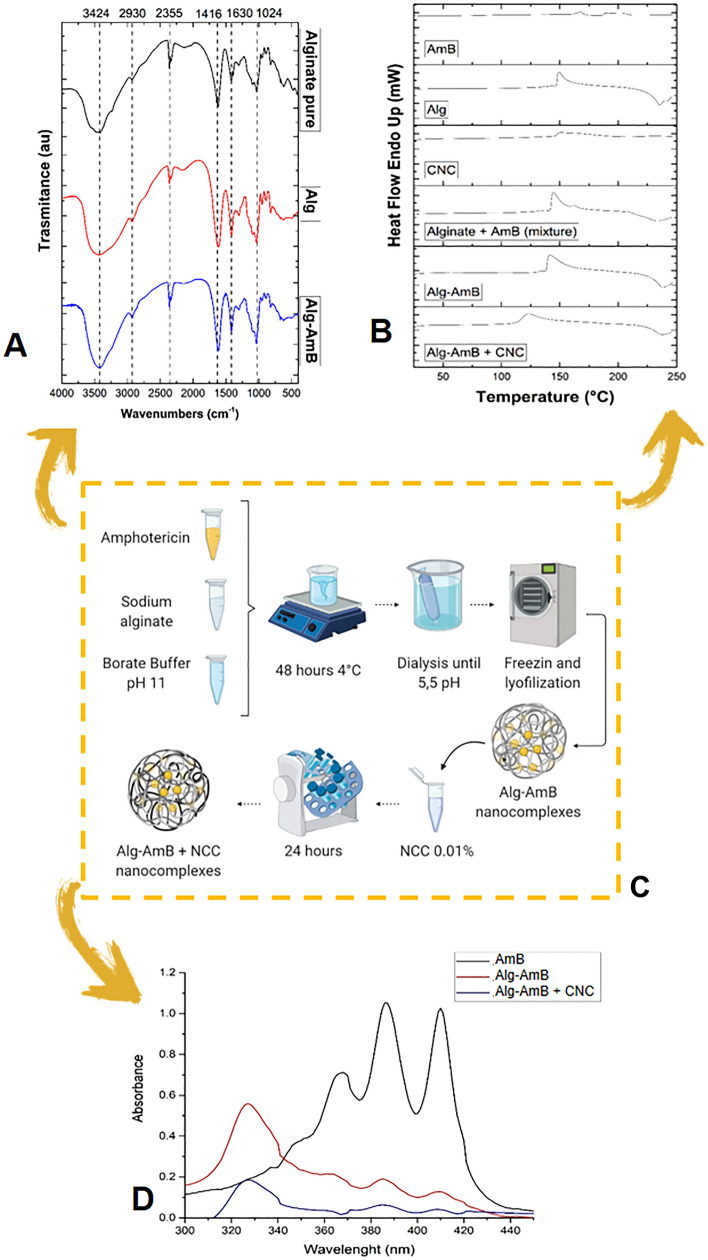
(**A**) Differential Scanning Calorimetry (DSC). (**B**) Infrared spectrum by Fourier transform (FTIR). (**C**) Design Production of Alg-AmB and Alg-AmB + CNC. (**D**) UV–Vis spectrometry. Note: A – DSC analysis of AmB, Alg, CNC, alginate + AmB (mixture), Alg-AmB and Alg-AmB + CNC. B – FTIR of pure alginate, Alg and Alg-AmB. D – UV–Vis spectrometry of the AmB, Alg-AmB and Alg-AmB + CNC.

Figure 1 shows the Differential Scanning Calorimetry (DSC) data for AmB, Alg, CNC, Alginate + AmB (physical mixture), Alg-AmB and Alg- AmB + CNC.

In DSC assay the AmB sample showed a peak in 167 °C degrees. For CNC sample the peak was at 151° C. The physical formation of AmB-alginate mixture showed peaks at 144 °C, referring to alginate, and at 161 °C referring to AmB. On the other hand, when Alg-AmB nanocomplexes were formed, DSC showed only one peak, at 141 °C, for alginate.

The DSC value for Alg-AmB + CNC sample showed a similar peak to Alg-AmB, but with a slightly lower temperature, at 123 °C. This indicates when CNC was added in the formulation, there was a decrease in the temperature.

Figure 1B shows the FTIR data of the samples of pure alginate, Alg and Alg-AmB. The wavenumbers band 3424 cm^−1^ presented in all samples refers to the O–H stretching vibrations of the alginate. The weak band at 2930 cm^−1^ represents the C–H stretching vibrations; the bands 1630 and 1416 cm^−1^ are asymmetrical and symmetrical stretching vibrations of the C=O and the COO- group. Furthermore, the band around 1024 cm^−1^ refers to the vibrations of the ring elongation C–O and C–O, with deformations of C–C–H and C–O–H^8,16^. In the Alg-AmB sample, the AmB wavenumbers bands were compared to data from the literature, showing the bands: OH stretching vibration (3434 cm^−1^), CH flexion vibrations and CH_3_ oscillation (1024 cm^−1^)^17^. The hypothesis is the other bands are overlapped by the alginate bands.

The Fig. 1D shows the UV–vis spectra of AmB, Alg-AmB nanocomplex and Alg-AmB + CNC.

In this study, AmB was firstly dissolved in 0.1 M borate buffer pH 11 and then diluted in water, in a monomeric state. The ratio Abs_349_/Abs_410_ was 0.36. Considering the Alg-AmB nanocomplex, the1st peak (327 nm) and the ratio between the peaks I and IV (Abs_327_/Abs_410_) was 4.38, showing AmB is in a supper-aggregated state in the formulation, which, as referred above, is associated to a low toxicity. Despite promoting a decrease in the absorbance intensity, CNC addition to the nanocomplex surface did not affect the supper-aggregated state of AmB since a ratio of 4.45 (Abs_327_/Abs_409_) was found.

### In vitro toxicity

**Figure 2:**
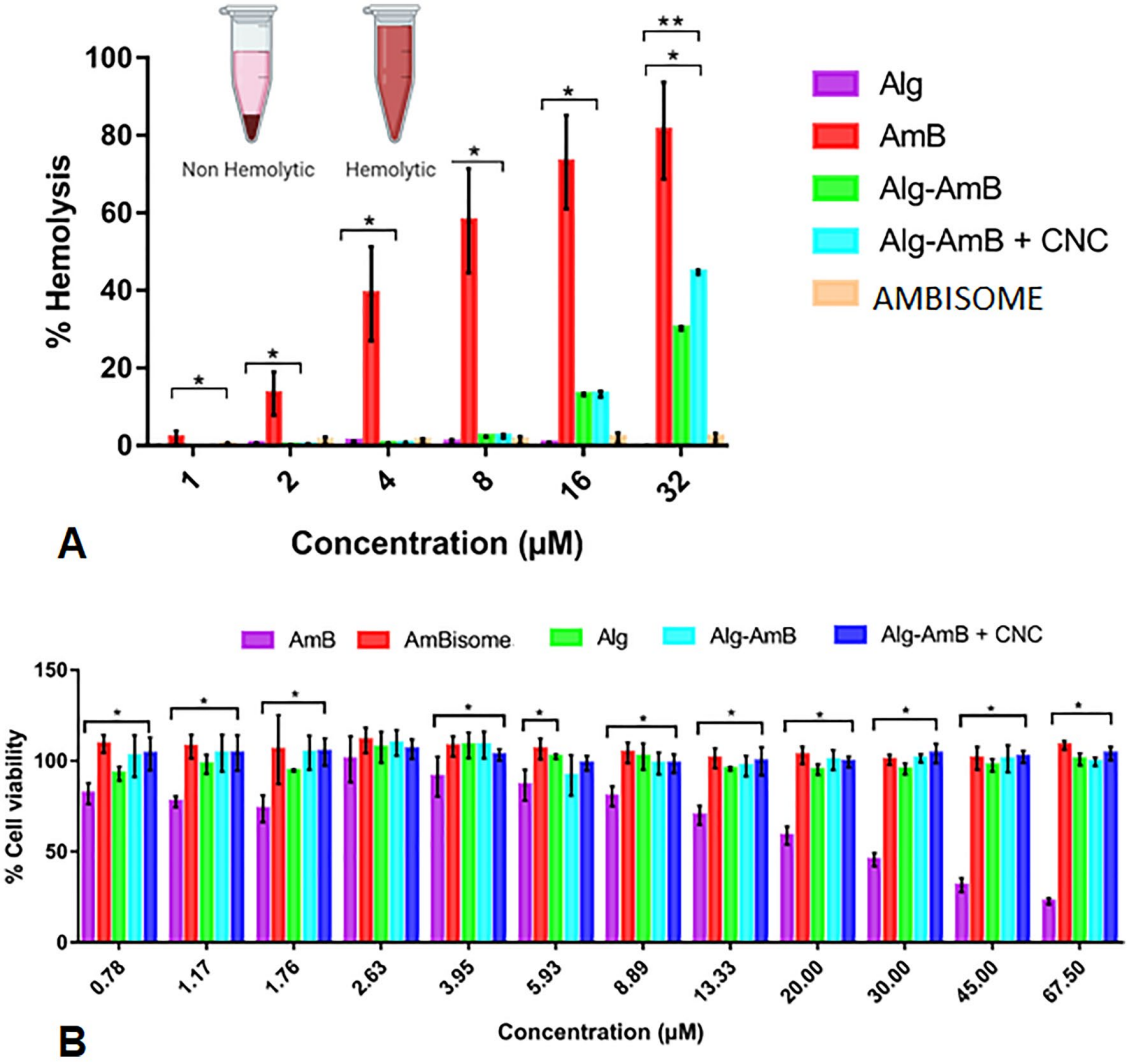
Percentage of hemolysis after treatment of red blood cells (**A**) Cytotoxicity of the renal cell line HEK293T (**B**) with Alg, Alg-AmB, Alg-AmB + CNC, AMBISOME and AmB. Note: A AmB (*) and Alg-AmB (**) were compared to the other groups by the Duncan test (p < 0.05). B – AmB (*) was compared to the other groups by the Duncan test (p < 0.05).

The hemolysis test was performed for AmB, Alg, Alg-AmB, Alg-AmB + CNC and AMBISOME, in concentrations of 1, 2, 4, 8, 16 and 32 µM (Fig. 2A).

The standard sample of Alg showed irrelevant values of hemolysis (lower than 2%), in all concentration. AmB induced hemolysis percentage between 1.97 ± 1.66% and 81.18 ± 12.50%. When Alg was added to the nanocomplexes formulation (Alg-AmB), it was observed a decrease in toxicity in 90% compared with the standard AmB. The hemolysis values for Alg-Amb was −0.08% to 30.25 ± 0.50%. For the Alg-Amb + CNC, the values were similar −0.3 ± 0.19% and 44.7 ± 0.50%. Even at high concentrations, nanocomplexes reduced the rate of hemolysis in comparison to standard AmB.

Regarding hemolytic porcetange of AMBISOME, data ranged from 0.34 ± 0.33% to 2.13 ± 1%. Although AmBisome's hemolysis values are lower than those found in the Alg-AmB formulation, Alg-AmB nanocomplexes had excellent results compared with free AmB. Hemolysis diminish in the sample due to the incorporation of AmB in Alg nanocomplexes shows the nanocomplexes are safe.

In Fig. 2B is presented the cytotoxicity data for AmB, Alg, Alg-AmB, Alg-AmB + CNC and AMBISOME. Alg showed cell viability between 93.01 ± 3.83% and 108.66 ± 7.04%. AmB data indicated concentration-dependent toxicity. On the other hand, the Alg-AmB nanocomplexes demonstrated significant less toxicity than free AmB. Cell viability was high, ranging between 97.16 ± 5.61% and 109.86 ± 7.06%.

For the Alg-AmB + CNC nanocomplexes, the cell viability values were similar to Alg-AmB CNC, ranging from 98.55 ± 5.06% to 106.40 ± 5.35%, demonstrating safety use of coating with CNC. Cell viability for AMBISOME was around 101.40 ± 6.23% to 111.29 ± 6.92%. When comparing the results of the commercial sample, AMBISOME, with Alg-AmB and Alg-AmB + CNC nanocomplexes, there was no significant difference among them, indicating both nanocomplexes, with or without CNC, are as safe as the commercial one.

## Discussion

In this work, alginate-amphotericin nanocomplexes (Alg-AmB) were developed aiming to improve the AmB water solubility with low toxicity. It was also evaluated the influence of CNC as a cover system to protect the system. When CNC is applied to cover nanocomplexes there was no CNC agglomeration. Similar result was observed in Soeiro et al.^35^. The authors established a new pathway to produce and apply the bacterial CNC.

Taheri and Mohammadi^18^ utilized cellulose nanocrystals to cover a hydroquinone system and they demonstrated an increase in particle size. The way to obtain smaller particles is important, because expanding the routes of administration. Systems with polymers nanoparticles of size around 200 nm are absorbed by the spleen, lung and liver^4^.

The nanocomplexes method is capable of producing smaller particles than ionotropic gelification method. Senna et al.^19^ produced particles by ionotropic gelation and observed the production of particles of 1.2 ± 0.34 mm. Compared to our method; these particles were about 3 times larger than nanocomplexes coated by CNC. One study with alginate particles containing miltefosine obtained by ionotropic gelation and emulsification method showed a polydispersity index of 0.43 ± 0.14^20^, similar to the data found in our study.

Comparatively in another study, the zeta potential of alginate particles obtained by ionotropic gelation showed negative values in the range of −15.7 ± 1.7 and −9.8 ± 1.2 mV. However, when adding chitosan in the formulation, there was a change in zeta potential due to the coating^21^.

AmB is characterized by two endothermic peaks in the crystalline form^22^. In our study, AmB standard showed peaks at 167 °C (melting point) and 198 °C, similar to the temperatures found by those authors. The DSC curves of CNC value were 151 °C an exothermic peak, probably a melting enthalpy. Similar result was found in our group study by Soeiro et al.^35^. Alginate showed an exothermic peak at 149 °C, which demonstrates the degradation of the biopolymer^23,24^. Vasconcelos et al. 2017^25^. reported peaks to CNC obtained by acid hydrolysis ranging from 140 to 195°C^25^, similar to our finding (peak at 151 °C). Regarding Alg-AmB nanocomplexes, we have described only one peak. The absence of AmB peak indicates there was a change from crystalline to amorphous state, demonstrating the formation and improvement of alginate and AmB nanocomplexes^26^.

In a study with alginate associated to the polymer poly [N-(2-hydroxypropyl) methacrylamide] and the drug camptothecin, the characteristic peak of camptothecin was absent due to the drug being compatible with the polymers and its high dispersion in the formulation^27^. About alginate sample, similar results were found by Silva-Carvalho et al., indicating the importance of using safe polysaccharides for the formation of nanocomplexes. Another examples can be observed in a study whose objective was to use CNC as a reinforcement for a film containing polylactic acid, concluding there was an increase in crystallinity^28^. George et al. reported the utilization of CNC associated with polymers and showed there was an increase in the parameters of glass transition, melting temperature, enthalpy and crystalline behaviour.

The degree of AmB aggregation can be determined by the ratio from the first to the fourth peak in the UV–Vis spectra, being a value of < 1 related to the monomeric form and a value of > 2 related to a supper-aggregated form^4,6,8,29^. In solution, AmB has three different states that affect its activity and pharmacokinetical characteristics, such as monomers, in which AmB is water soluble normally associates with ergosterol in fungal and protozoan cell membranes; oligomers, a state in which small water aggregates are toxic towards host cells and present very low solubility; and poly-aggregates, that also referred as supper-aggregates present low in vitro and in vivo toxicity^6,8^.

Fungizone® and AMBISOME are drugs with low toxicity found in the pharmaceutical market. Due to the super-aggregated state of AmB, specified by ratio values of 2.9 and 4.8 (Alg-AmB and Alg-AmB + CNC, respectively), our nanocomplexes have similar data in relation to commercial formulations^7,30^.

The hemolysis test is important in studies with nanocomplexes or nanoparticles containing AmB once there is evidence of hemolytic anemia caused by the use of free AmB^31,32^. AmB has a high hemotoxicity even at low concentrations, due to the aggregated conformation^31^. Conjugates and ionic cross-linked polymeric nanoparticles of AmB with alginate show less hemotoxicity and greater hemocompatibility than free AmB, possibly by the protective effect from polysaccharide and by the change in conformation of the super-aggregated state^4,8^.

The importance of cytotoxicity test with this cell strain is due to the nephrotoxicity associated with free AmB^7^. Therefore, it is important to understand the behaviour of nanocomplexes in this type of cells. The non-toxicity and the capacity for cell proliferation observed to alginate experimentation have already been described^4^. In a study, AmB solution at 15.6 µg/mL was able to induce death in renal cells^17^. On the other hand, Ravichandran and Jayakrishnan, 2018 tested AmB and alginate conjugates to verify cytotoxicity and observed a decrease in toxicity compared to free AmB.

Although there are studies reporting reduction in hemo and cytotoxicity of amphotericin, our work proposes a simplified methodology, with a cost–benefit and achieves the same objectives by complex and high cost methodologies. Therefore, in addition to our material being as safe as the standard medicine (AMBISOME). Our work results demonstrated the applicability of nanocomplex as AmB carrier, similarity results was found in Silva-Carvalho et al. work. However our nanocomplexes was produced with alginate and covered with CNC.

## Conclusion

In our study, the alginate nanocomplexes with AmB decreased its hemolytic and cytotoxic effect. In addition of this, Alg-AmB nanocomplexes were covered by CNC and the improve of pharmaceutical characteristics of the system was observed. There was observed advantages attributed to the developed formulation, which can be considered a cost benefit, non-toxic and safe as the commercial one. These findings have an important relevance, once when developing nanocomplexes, we can direct the formulation. It means that nanocomplexes can be formulate with specific action sites. And, when the CNC is apply as cover, can give an extra protection to the nanocomplexes and it can be apply in different administration via, such as oral.

## Methodology

### Reagents

Amphotericin B (AmB, molecular weight of 924.08 g/mol) powder from *Streptomyces* sp., resazurin Sodium salt, sodium alginate, triton X-100, sodium tetraborate decahydrate were purchased from Sigma-Aldrich (Missouri, USA). Dulbecco's Modified Eagle Medium (DMEM), fetal bovine serum (FBS) and penicillin–streptomycin were obtained from Merck Millipore (Massachusetts, USA). Roswell Park Memorial Institute (RPMI) 1640. Glutamax supplemented medium and L-glutamine (GlutaMAX™-I) was purchased from Gibco (Massachusetts, USA). Dimethylsulfoxide (DMSO ATCC® 4-X™) solution for cell culture was acquired from American Type Culture Collection (ATCC, Virginia, USA). Dialysis tubing with a molecular weight cut-off of 1000 Da was obtained from Orange Scientific (Braine-l'Alleud, Belgium). AMBISOME was kindly provided by Gilead Sciences.

### Bacterial cellulose nanocrystals (CNC) production

The bacterial cellulose membranes (BNC) were produced by *Komagataeibacter xylinus* (ATCC 53582), in Hestrin&Schramm broth, according to Jozala et al.^33^. The BNC were processed in a mechanical treatment with ultra-turrax and high-pressure homogenizer; and after, in a enzymatic treatment with cellulase for 72 h, to obtain CNC. The CNC were separated by centrifugation and filtration following the methodology developed by our research group (Soeiro et al.)^35^.

### Preparation of alginate-amphotericin B (Alg-AmB) nanocomplexes

The Alg-AmB nanocomplexes were prepared according to Silva-Carvalho et al.^5^ with modifications. In our formulation, borate buffer (pH 11.0), sodium alginate and amphotericin B were utilized. The final concentration of AmB was 2.5 mg/mL in the solution. The nanocomplexes formulation were described in Table 1.

The nanocomplexes samples were kept under agitation at 4 °C for 48 h, inside temperature laboratory chamber, without interference from light. After that, the sample was run in dialysis membrane from 12,000 to 14,000 KDa. The membrane with sample was immersed in 5 L of distilled water under agitation at 4 °C, without light interference, for 30 h. During the dialysis process, the water was changed three times, until reaching pH value in the range of 5.5–5.7. Then, the samples were collected, stored at −80 °C for 24 h, and lyophilized for 72 h. The nanocomplexes yield was 73.72%.

### Nanocomplexes covered by CNC

Alg and Alg-AmB nanocomplexes were covered with CNC according to Soeiro et al.^35^. Then, an amount of 10 mg of lyophilized nanocomplexes were dispersed in 10 mL water. After that, 1 mL of the nanocomplexes solution was added into 1 mL of 0.01% CNC suspension. The sample was kept on rotatory shaker (20 rpm) at 25 °C for 24 h.

### Nanocomplexes physico-chemical characterization

The particles were characterized by the techniques of index of differential scanning calorimetry (DSC), size, polydispersity, zeta potential, infrared spectrometry by Fourier transform (FTIR) and UV–Vis spectrometry.

For the size, index of polydispersity and zeta potential were measured by the Zetasizer equipment (ZEN3600). The analysis was performed for six times, under an angle of 173º, at 25 °C.

The nanocomplexes stability was analysed by Differential Scanning Calorimetry (DSC). The characterization was performed by the DSC 6000 equipment (PERKIN ELMER | STEC INSTRUMENTS) in a nitrogen atmosphere with a flow of 20 mL/min, in the heating range of 25–250 °C with a heating rate of 10 °C/min. The test was re-performed with the samples AmB, Alg nanocomplexes, CNC, alginate + AmB (physical mixture without nanocomplexes process), Alg-AmB nanocomplexes and Alg-AmB nanocomplexes + CNC.

The chemical groups characterization was carried out by spectrometry in FTIR. The samples pure alginate, Alg nanocomplexes and Alg-AmB nanocomplexes were analysed by KBr tablets technique. The technique was performed utilizing 2 mg of the sample and 200 mg of the KBr. The FTIR spectra were obtained in the range of 4000–400 cm^−1^ in the Bruker Alpha II equipment, with a resolution of 4 cm^−1^ and 12 scans.

In addition, the Alg nanocomplexes and Alg-AmB nanocomplexes were dispersed in distilled water (1 mg of sample/mL) and analysed by UV–Vis spectroscopy. The analyses were performed in the 300–450 nm range using a UV–Visible spectrophotometer (JASCO V-560) with 5 nm resolution and scanning speed of 400 nm/min.

### In vitro toxicity

The in vitro toxicity analysis were run to evaluate the safety of the nanocomplexes compared with a commercial product, AMBISOME. AMBISOME is an injectable drug containing with a liposomal formulation. Its prescription is mentioned in several articles due to low toxicity^6,7,34^.

### Hemolysis test

The hemolysis protocol and the animal's blood were performed according Silva-Carvalho et al.^5^. The blood sample was collected in EDTA tube from a healthy dog with the owners' consent. The blood sample was centrifuged for 10 min, at 4 °C and 1200 G. Then, the supernatant was discarded and the red blood cells were resuspended in phosphate buffer solution (pH 7.4).

The 450 µL of red blood cells at a concentration 1 × 10^8^ cells / mL were placed in a 48-well plate in contact with 50 µL of the samples of AmB, Alg, Alg -AmB nanocomplexes, Alg-AmB nanocomplexes + CNC and AMBISOME. The samples were at concentrations of 1, 2, 4, 8, 16 and 32 µM. The 48-well plate was incubated under agitation for 30 min, at 37 °C and 120 rpm.

After the time, the solutions were collected and centrifuged for 10 min (4° C, 1200 G). The supernatants were collected and analyzed by UV–Vis spectrophotometry, with absorbance at 540 nm. The complete hemolysis was considered when hemoglobin was released with 1% triton X-100 (positive control).

### Cytotoxicity

Human Embryonic Kidney (HEK) cell line was selected to perfume citotoxity due to the toxicity of AmB in renal cells. The HEK 293 T monolayer (1 × 10^4^ cell/well) was incubated for 24 h (37 °C in a 5% CO_2_ atmosphere) with AmB, Alg, Alg-AmB, Alg-AmB + CNC and AMBISOME at concentrations of 0.78, 1, 17, 1.76, 2.63, 3.95, 5.93, 8.89, 13.33, 20, 30, 45 and 67.5 μM. After the incubation period, 10% (v / v) of a 2.5 mM resazurin solution was added to each well and the plates were incubated again under the same conditions as above for 4 h.

Fluorescence was measured (λ 560/λ 590) in a SpectraMAX GeminiXS microplate reader (Molecular Devices LLC, California, USA). The results were expressed as mean percentage ± SD of viable cells in relation to the positive control (condition considered to be 100% viable cells).

### Statistical analysis

Data from toxicity tests were expressed as mean ± standard deviation. Analysis of Variance (ANOVA) followed by the Duncan test were used to verify differences among treatment protocols, and p values < 0.05 were considered significant. Results were analyzed using Statistica® v. 8.0 (Dell, Round Rock, TX, USA) and GraphPad Prism® v. 6.0 (San Diego, CA, USA).
